# The Nexus of the Dark Triad Personality Traits With Cyberbullying, Empathy, and Emotional Intelligence: A Structural-Equation Modeling Approach

**DOI:** 10.3389/fpsyg.2021.659282

**Published:** 2021-06-04

**Authors:** Estelle C. Schade, Martin Voracek, Ulrich S. Tran

**Affiliations:** Department of Cognition, Emotion, and Methods in Psychology, School of Psychology, University of Vienna, Vienna, Austria

**Keywords:** cyberbullying, Dark Triad, emotional intelligence, empathy, Machiavellianism, narcissism, psychopathy, structural equation modeling

## Abstract

This study set out to elucidate the complex suite of associations between the Dark Triad personality traits (Machiavellianism, narcissism, and psychopathy), emotional intelligence, empathy, and cyberbullying, as the respective findings regarding this topic have been inconsistent. Studies preponderantly have relied on abbreviated Dark Triad measures that do not differentiate between its lower-order facets. Further, most extant studies have exclusively been based on female psychology undergraduates and have not accounted for known sex differences on the Dark Triad traits and cyberbullying, or for negative associations between cyberbullying and age. Therefore, this nexus of interrelations was investigated in a diverse community sample (*N* = 749). A structural equation-modeling approached was used to examine predictors of cyberbullying and to test for mediating relationships between lower-order Dark Triad facets and emotional intelligence and empathy. Multigroup models were applied to test for sex-specific patterns. Empathy did not predict cyberbullying, whereas emotional intelligence partly mediated the Dark Triad associations with cyberbullying among both sexes. Sex-specific patterns in the associations between Dark Triad traits and cyberbullying were particularly observed for the grandiose and vulnerable narcissism facets. Emotional intelligence appeared to buffer effects of grandiose narcissism on cyberbullying. Future research could fruitfully explore cyberbullies’ profiles regarding primary and secondary psychopathy, sex differences in narcissism, and buffering effects of emotional intelligence. Further improvements regarding the measurement of dark personality traits are indicated as well.

## Introduction

Bullying is a proactive form of aggression (Boulton and Smith, 1994; Sutton et al., 2001) which has been in the focus of research for quite some time, whereas cyberbullying is a topic which only surfaced with the increasing spread of the internet and social media. Tokunaga (2010, p. 278) defines cyberbullying as “any behavior performed through electronic or digital media by individuals or groups that repeatedly communicates hostile or aggressive messages intended to inflict harm or discomfort on others”, which thus includes online stalking and harassment (Lowry et al., 2016).

Depending on the breadth of the definition used, prevalence rates of cyberbullying vary between 5 and 65% (Kowalski et al., 2018). Possible consequences are emotional and psychosocial distress (Gillespie, 2006), including depression, alexithymia, insomnia, somatic symptoms, substance use, depression, avoidance, and fear (Aboujaoude et al., 2015). The damage resulting from cyberbullying depends on its frequency, length, and severity: less regular and shorter cyberbullying periods lead to less severe consequences (Tokunaga, 2010).

It has been estimated that 1–44% of the population are perpetrators of cyberbullying (Kowalski et al., 2018). Perpetrators often show behavioral problems, as well as hyperactivity and substance abuse (Aboujaoude et al., 2015). Moreover, anonymity implies lower costs, which has been associated with an increase in cyberbullying behavior. Women see more costs and less benefits than men; hence, they are less prone to exert cyberbullying behavior (Lowry et al., 2016). Furthermore, age seems to have an impact on cyberbullying as well, as older individuals have more difficulties, less routine, and less motivation to use social media in general, but also to use it in a malevolent way (Ionut-Dorin, 2017).

### Emotional Intelligence and Empathy

When it comes to understanding cyberbullying, two important factors appear to be emotional intelligence and empathy. Emotional intelligence (Salovey and Mayer, 1990) is “the ability to perceive accurately, appraise, and express emotion; the ability to access and/or generate feelings when they facilitate thought; the ability to understand emotion and emotional knowledge; and the ability to regulate emotions to promote emotional and intellectual growth” (Jung and Yoon, 2012, p. 370). Specifically, trait emotional intelligence is a self-perceived aspect of personality, measured through self-reports, regarding the way an individual responds to emotional cues. In contrast, ability emotional intelligence involves cognitive processes and actual emotional abilities and is measured with maximum-performance tests (Mavroveli et al., 2007; Baroncelli and Ciucci, 2014).

Emotional intelligence has some parts in common with empathy (Keskin et al., 2016). Empathy is the ability to understand and experience other people’s emotions (Schokman et al., 2014). Thus, it needs to be differentiated from perspective taking and sympathy. Feeling sad for another person, because that other person is sad, is empathy (also often called ‘affective empathy’), whereas understanding why that other person is sad is perspective taking (also often called ‘cognitive empathy’), and feeling concerned for that person’s difficult situation is sympathy.

Ineffective or inappropriate reactions, difficulties cultivating positive affect, and not diminishing the strain of negative affect appear to elevate the risks of bullying or being bullied (Schokman et al., 2014). In contrast, high emotional intelligence and high empathy appear to be linked to decreased cyberbullying behavior (Baroncelli and Ciucci, 2014; Ang et al., 2017). However, perspective taking and emotional intelligence also can be useful tools to effectively manipulate or bully others (Sutton et al., 2001; Baughman et al., 2012; Vonk et al., 2015; Davis and Nichols, 2016). Empathy and emotional intelligence might thus act as mediators of the associations between aversive and antagonistic personality traits, like those of the Dark Triad, with cyberbullying behavior.

### The Dark Triad

The Dark Triad of personality (Paulhus and Williams, 2002) subsumes three aversive personality traits, namely, Machiavellianism, narcissism, and psychopathy, which have been linked to a dark personality core and to low empathy, low emotional intelligence, and bullying (Jones and Figueredo, 2013; Bertl et al., 2017; Muris et al., 2017). Even though sometimes understood as discrete typologies, the Dark Triad traits, along with their underlying dark core, most likely are dimensional constructs (Tran et al., 2018). There are known sex differences on all Dark Triad traits, with men consistently scoring higher than women (Muris et al., 2017).

#### Psychopathy

Psychopathy is characterized by a lack of empathy, sympathy, love, remorse, shame, guilt, and superficial emotions (Cleckley, 1988; Hart et al., 1992). Individuals high in psychopathy do not consider the welfare of others and do not refrain from harming other people or disobeying moral conventions if they are not in line with their own goals or wishes. In fact, their acceptance of moral rules is only an illusion (Matthews, 2014). Overall, psychopathy seems to predict proactive and reactive aggression (Knight et al., 2018), bullying (Baughman et al., 2012), cyberbullying (Goodboy and Martin, 2015), cyberaggression (Pabian et al., 2015), and emotional manipulation (Nagler et al., 2014).

Psychopathy can further be differentiated into primary and secondary psychopathy (Karpman, 1941). Primary psychopathy appears to be a heritable personality trait linked to callousness, empathy deficits, and lack of fear (Karpman, 1941; Hicks et al., 2012). Individuals high in primary psychopathy show lower degrees of mental and physical stress, reduced physiological reactions, perception, and processing of social and emotional cues, less affective interference during moral appraisal and choice-making, and an increased ability to manipulate, deceive, and persuade others (Yildirim and Derksen, 2013). Primary psychopathy is not only characterized by low empathy, but also by lower trait emotional intelligence (Vonk et al., 2015; Szabó and Bereczkei, 2017). Yet, individuals high in primary psychopathy still possess the ability to function well in society. They take risks and navigate social situations in a planned, strategic, foreseeable, controlled way, with adequate behavior, in order to reach their goals and to succeed (Gao and Raine, 2010; Poythress and Hall, 2011).

Secondary psychopathy is believed to be a consequence of trauma and disturbed conscience resulting therefrom, comprised of the belief that humankind and the world are contentious and bad (Karpman, 1941). A more recent study by Hicks et al. (2012) found that it does also have a heritable component that leads to a greater exposure to environmental risk factors, especially for men. A high-functioning temperament combined with negative social experiences or abuse can lead to the development of characteristics very similar to those of primary psychopathy (Yildirim and Derksen, 2013). However, only secondary psychopathy shows associations with alexithymia (Lander et al., 2012). Yet, individuals high in secondary psychopathy are still able to share an emotional connection with others and feel negative affect, such as angst and culpability (Karpman, 1941), even though they might not be able to correctly describe it and lack emotional intelligence (Vonk et al., 2015; Szabó and Bereczkei, 2017). Illicit and hostile actions of individuals high in secondary psychopathy have been reported to be less strategic and more impulsive than those of individuals high in primary psychopathy (Skeem et al., 2007).

#### Narcissism

Narcissism is “the pursuit of gratification from vanity or egoistic admiration of one’s own attributes” (Muris et al., 2017, p. 184) and can exert various negative influences on relations with other individuals (Campbell et al., 2010). There are two distinct forms of narcissism in the general population, namely, grandiose and vulnerable narcissism (Jauk and Kaufman, 2018), which share antagonistic traits, but are quite distinct regarding their interactions, affective experiences, and the nature of behavioral reactions shown (Miller et al., 2011).

Individuals high in grandiose narcissism show high levels of adaptive and maladaptive behavior, making them pretentious, conceited, and exhibitionistic (Dickinson and Pincus, 2003). Grandiose narcissism subsumes lower-order factors of entitlement and exhibitionism, leadership, exploitativeness, grandiose fantasies, and entitlement rage (Miller et al., 2011). The leadership facet is considered an adaptive aspect of the construct, as it appears to be linked to positive outcomes (e.g., self-esteem and less internalizing psychopathology); other facets are clearly more maladaptive, concerning their correlates (Ackerman et al., 2011). Grandiose narcissism is more prevalent among men, as men feel more entitled and powerful in general (Grijalva et al., 2015). Individuals high in grandiose narcissism are very focused on social status, dominance, success, and connections, whereas much less so on intimacy (Jauk and Kaufman, 2018). Consequently, they are rather unfriendly, selfish, and deceitful, and oftentimes exhibit blatant aggressive behavior (Miller et al., 2011; Wallace et al., 2016), especially in reaction to criticism or threats to their image or self-esteem (Hyatt et al., 2018). Regarding their emotionality, individuals high in grandiose narcissism seem to be less afflicted by fear, depression (Jauk and Kaufman, 2018), psychological distress, or negative emotions in general (Miller et al., 2011). Rather, they show high mental flexibility and stable mental health. However, they do not experience particularly positive affect either (Jauk and Kaufman, 2018).

Individuals high in vulnerable narcissism show low levels of adaptive and high levels of maladaptive behavior, meaning that they are introverted and fearful, but entitled and manipulative at the same time (Dickinson and Pincus, 2003). They are neurotic, have a tendency for avoidant behavior, and low self-esteem (Jauk and Kaufman, 2018). Correspondingly, vulnerable narcissists display an abnormal attachment style defined by fear (corresponding to higher levels of psychological distress), antagonism, depression, and mistrust (Miller et al., 2011). Thus, regarding their emotionality, individuals high in vulnerable narcissism experience little positive affect and high levels of negative emotions (Jauk and Kaufman, 2018). They have a negative world and person view, which is associated with an impaired regulation of emotions and negative relational schemata, probably resulting from traumatic experiences (Rogosch and Cicchetti, 2004). Individuals high in vulnerable narcissism can be very hostile and tend to react with embarrassment, but also with anger (Hyatt et al., 2018), as aggression toward others also serves as a positive reinforcement of their self-image (Parton and Ent, 2018). Importantly, their aggression rarely is in the form of verbal aggression, as they care about others’ opinions, and more often is executed in a concealed way (Okada, 2010).

Both vulnerable and grandiose narcissism predict proactive and reactive aggression (Knight et al., 2018) and bullying (Baughman et al., 2012; Fanti and Kimonis, 2012), and grandiose narcissism predicts also emotional manipulation (Nagler et al., 2014). Interestingly, grandiose narcissism does not seem to uniquely predict cyberbullying (Gibb and Devereux, 2014; van Geel et al., 2017). Positive associations between grandiose narcissism and emotional intelligence have been reported (Vonk et al., 2015; Szabó and Bereczkei, 2017). However, among men, ability trait emotional intelligence and grandiose narcissism appear to be negatively correlated (Jauk et al., 2016).

#### Machiavellianism

Machiavellianism is characterized by “a duplicitous interpersonal style, cynic disregard for morality, and focus on self-interest and personal gain” (Muris et al., 2017, p. 184), but is not linked to a specific clinical diagnosis (Beller and Bosse, 2017). The characteristics of Machiavellianism can be distinguished into cognitions, desires, and behavior. Cognitions encompass self-absorption, negative world views, and the tendency to plan and scheme ahead. Regarding their desires, individuals high in Machiavellianism are focused on self-promotion and self-protection, status and dominance, and impulse regulation to attain their goals. As a result, their behavior tends to be abusive, impassive, hostile, self-centered, and manipulative (Rauthmann and Will, 2011).

Machiavellianism has been linked to lower empathy and higher callousness, but also to higher emotional intelligence (Monaghan et al., 2018). However, other studies have reported negative associations with trait emotional intelligence (Ali et al., 2009), and that individuals high in Machiavellianism might have trouble with (or less incentives for) processing emotions in general (Czibor et al., 2017). Men displaying higher trait emotional intelligence have been reported to show less Machiavellian behavior and delinquency; in contrast, higher trait emotional intelligence in women is linked to more Machiavellian behavior (Bacon and Regan, 2016). Markedly, women high in Machiavellianism tend to use emotional manipulation, especially toward close friends, to achieve their goals (Czibor et al., 2017).

There are associations between Machiavellianism and bullying (Baughman et al., 2012) and emotional manipulation (Nagler et al., 2014). However, findings related to cyberbullying have been inconsistent. Whereas a number of studies have reported positive associations between Machiavellianism and cyberbullying (Wang et al., 2016; Kircaburun et al., 2018), others did not yield evidence for specific contributions of Machiavellianism in predicting cyberbullying (Gibb and Devereux, 2014; van Geel et al., 2017).

### Goals of the Present Study

The Dark Triad traits have antagonistic interpersonal behavior and negative psychosocial effects in common (Muris et al., 2017). Specifically, Machiavellianism and psychopathy share a tendency toward malevolent behavior (Lilienfeld and Andrews, 1996), insincerity, and unfairness (Muris et al., 2017). Narcissism differs the most from the other Dark Triad traits, as it additionally entails a vulnerable component, based on self-protection, and is linked to avarice. It has also been located in a separate, vulnerable, Dark Triad, together with secondary psychopathy and borderline personality disorder (Miller et al., 2010). However, narcissism does display malice as well, which explains its association with a common dark personality core (Miller et al., 2010; Veselka et al., 2014; Muris et al., 2017). Even though the Dark Triad traits can be studied as one dark core, each trait also shows unique characteristics which are worthy detailed investigation.

Cyberbullying is a problem in contemporary society and seems to be linked to sex, age, emotional intelligence, empathy, and the Dark Triad traits. While there are several studies (Baughman et al., 2012; Lowry et al., 2016; Ang et al., 2017; Ionut-Dorin, 2017; Muris et al., 2017) on these topics, conclusions have often been mixed, if not contradictory. There are known sex differences in the Dark Triad traits and in cyberbullying, and negative associations of cyberbullying with age. However, studies in the field of the Dark Triad have mainly been based on female psychology undergraduates. Moreover, many studies have only used brief measures of the Dark Triad [like the Dark Triad Dirty Dozen, Jonason and Webster, 2010; or the Short Dark Triad (SD3), Jones and Paulhus, 2014], which are less differentiated than longer scales (i.e., lack lower-order facets) and do not measure all dimensions of the Dark Triad traits (Muris et al., 2017). For these reasons, more comprehensive measures should be used, and research should be based on samples more balanced with regards to participant sex and more diverse with regards to participant age.

**FIGURE 1:**
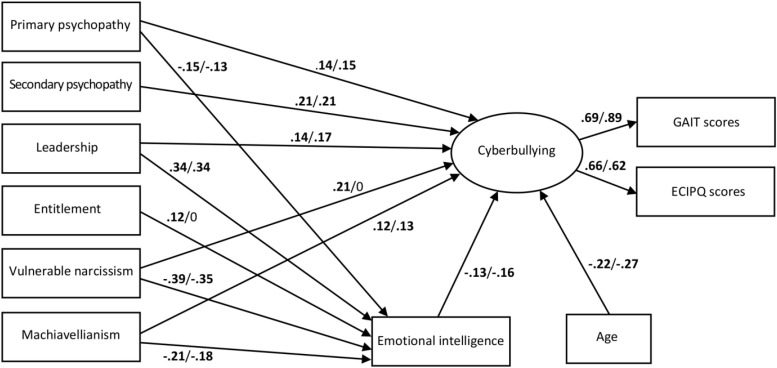
Final multigroup path model. Numbers are standardized coefficients (**left:** men, **right:** women; all *p*s < 0.05, except for coefficients of 0), which were constrained to equality (on their unstandardized scale) across groups (due to differences in dispersion, standardized coefficients may still differ between groups). Coefficients of 0 were set to 0 in the analysis and not freely estimated from the data. GAIT, global assessment of internet trolling; ECIPQ, European Cyberbullying Intervention Project Questionnaire – Subscale Aggression.

Currently, the nexus of associations between emotional intelligence, empathy, the Dark Triad, and cyberbullying awaits further elucidation. Therefore, we examined the associations between these traits in a large and diverse community sample, utilizing comprehensive measures of the Dark Triad traits and pursuing a structural equation-modeling approach. Specifically, we controlled for possible age effects and examined whether the respective associations between personality traits were similar among men and women, using multigroup analysis (see Figure 1 for a visualization of the full path model).

We expected that (1) cyberbullying correlates positively with the Dark Triad traits and (2) negatively with empathy and emotional intelligence. We further hypothesized that (3) empathy and emotional intelligence mediate the positive associations between the Dark Triad traits and cyberbullying.

## Materials and Methods

### Participants and Procedure

In total, data of 749 participants were collected from German-speaking communities in Austria and Germany between March and June of 2018. Of the participants, three indicated “other” regarding their sex and thus were excluded from further analysis, as multigroup analysis with such a small group was not possible (there were further 3 missing values). This resulted in a final sample of 743 individuals for analysis (44% from Austria, 52% from Germany, 4% from other European countries; and 11 missing values), of which 54% were women and 46% men. Median age was 25 years, with an age range of 19–81 years and an interquartile range of 22–47 years. Twelve percent of the sample had completed primary education, 20% lower secondary education, 44% upper secondary education, and 23% tertiary education (4 missing values); 40% of participants were students (9 missing values). Participants provided informed consent and did not receive any compensation for their participation. Participation was voluntary and anonymous.

### Materials

All self-report instruments were administered in German-language forms. German versions of the GAIT, HSNS, MACH^∗^, and SRP-III were produced using the parallel-blind translation technique (Behling and Law, 2000). Sample reliability coefficients (Cronbach α) are provided Table 1.

**TABLE 1:** Intercorrelations and internal consistency (Cronbach α) of all study variables in the total sample, and means and standard deviations for men and women (alongside effect sizes, Cohen *d*, for sex differences).

Scale or subscale	(1)	(2)	(3)	(4)	(5)	(6)	(7)	(8)	(9)	(10)
(1) Emotional intelligence	0.82									
(2) Empathy	–0.06	0.79								
(3) Primary psychopathy	–0.07	−0.22***	0.65							
(4) Secondary psychopathy	–0.02	–0.06	0.59***	0.86						
(5) Leadership	0.28***	–0.07	0.38***	0.35***	0.89					
(6) Entitlement and exhibitionism	0.15***	0.04	0.42***	0.40***	0.73***	0.83				
(7) Vulnerable narcissism	−0.38***	0.24***	0.13***	0.15***	0.12***	0.27***	0.66			
(8) Machiavellianism	−0.29***	−0.17***	0.26***	0.14***	0.03	0.08*	0.24***	0.70		
(9) Cyberbullying (GAIT)	−0.18***	–0.07	0.40***	0.44***	0.28***	0.28***	0.21***	0.27***	0.76	
(10) Cyberbullying (ECIPQ)	−0.13**	0.01	0.28***	0.30***	0.19***	0.21***	0.17***	0.16***	0.49***	0.83
(11) Age	0.05	−0.12**	−0.20***	−0.34**	−0.18***	−0.36***	−0.10***	–0.03	−0.32***	−0.27***
Men *M*	153.18	34.51	31.16	42.96	32.28	16.04	26.62	15.75	6.10	1.17
*SD*	16.35	7.01	5.83	12.34	9.31	5.59	5.65	5.13	2.73	1.93
Women *M*	151.87	39.03	27.57	35.84	28.49	14.13	26.90	13.92	4.71	0.96
*SD*	17.59	6.42	5.24	10.62	9.71	5.41	5.40	4.60	1.61	1.77
Cohen *d*	0.08	−0.68***	0.65***	0.62***	0.40***	0.35***	–0.05	0.38***	0.63***	0.12

GAIT, global assessment of internet trolling; ECIPQ, European Cyberbullying Intervention Project Questionnaire – Subscale Aggression. Sample internal consistency coefficients (Cronbach α) are presented on the main diagonal. For Cohen d, the positive sign indicates higher values for men, compared to women. *p < 0.05, **p < 0.01, ***p < 0.001.

#### Trait Emotional Intelligence Questionnaire – Short Form (TEIQue-SF; Petrides and Furnham, 2006)

The TEIQue-SF (German form provided at https://psychometriclab.com/translations-of-teique/) comprises 30 items and measures global trait emotional intelligence, comprising the factors Well-being, Self-control, Emotionality, and Sociability (Petrides, 2009). Item responses were made on 7-point scales (1: *completely disagree*; 7: *completely agree*).

#### Interpersonal Reactivity Index (IRI; Revised German Version: Paulus, 2009)

The IRI, originally developed by Davis (1980), is based on an operational definition of empathy and measures sympathy, perspective taking in general, and empathy. For this study, the revised (16-item) German version of the questionnaire was used. This version shows better reliability and validity (Paulus, 2009). The response range was from 0 (*never*) to 4 (*always*) on a 5-point scale.

#### Narcissistic Personality Inventory-15 (NPI-15; German Translation: Spangenberg et al., 2013)

The NPI-15 is an abbreviated version of the NPI-40 (Raskin and Hall, 1979) and measures grandiose narcissism with the two subscales Leadership (which is considered an adaptive aspect of grandiose narcissism) and Entitlement and Exhibitionism (considered as a maladaptive aspect). The German NPI-15 has a strong unrotated first factor and can thus be considered to measure a unified construct (see Spangenberg et al., 2013). Yet, we still used the two subscale scores for analysis, following recommendations of Ackerman et al. (2011) to investigate narcissism at the subscale level. The forced-choice response format was modified to a 6-point Likert scale, ranging from 1 (*completely disagree*) to 6 (*completely agree*).

#### Hypersensitive Narcissism Scale (HSNS; Hendin and Cheek, 1997)

The HSNS measures vulnerable narcissism in the general population. Its 10 items are answered on 5-point scales (1: *very uncharacteristic or untrue, strongly disagree*; 3: neutral middle; 5: *very characteristic or true, strongly agree*).

#### MACH^∗^ (Rauthmann, 2013)

The MACH^∗^ is a short version of the MACH-IV (Christie et al., 1970), comprised of the five items with the best validity for measuring Machiavellianism according to item response theory analyses. Responses are given on a 6-point scale, ranging from −3 (*strongly disagree*) to +3 (*strongly agree*), with no neutral middle category.

#### Self-Report Psychopathy Scale-III (SRP-III; Paulhus et al., 2016)

The revised form of the SRP-III measures psychopathy, with 31 items on 5-point scales, ranging from 1 (*strongly disagree*) to 5 (*strongly agree*), was used. The items belong to four subscales: two facets (Interpersonal and Affective) measuring primary psychopathy, and two further facets (Lifestyle and Antisocial) measuring secondary psychopathy. Based on exploratory factor-analytic results, Item 4 was removed because of its weak loading.

#### Global Assessment of Internet Trolling (GAIT; Buckels et al., 2014)

The GAIT measures internet trolling with four items on a 5-point scale, ranging from 1 (*completely disagree*) to 5 (*completely agree*). Both the GAIT and the ECIPQ were used as indicators of cyber-bullying.

#### European Cyberbullying Intervention Project Questionnaire – Subscale Aggression (ECIPQ; Del Rey et al., 2015; German Translation: Schultze-Krumbholz and Scheithauer, 2011)

The cyber-aggression subscale of the ECIPQ comprises 11 items assessing the frequency of different aversive online behaviors (i.e., cyberbullying). Answers are given on 5-point scales, ranging from 0 (*no*) to 4 (*yes, more than once a week*), and these responses pertain to whether or not the participant showed the specified behavior during the past 2 months. Both the GAIT and the ECIPQ were used as indicators of cyber-bullying.

### Data Analysis

Structural equation modeling (SEM) was used to examine the associations between the predictors, their expected mediators, and cyberbullying, using the two cyberbullying scores (GAIT and ECIPQ) to obtain a latent variable of cyberbullying in the model. Before fitting the model, data were winsorized at 2.5 standard deviations below and above their respective scale means to minimize possible distorting effects of outlying data points (this entailed no exclusion of participants). The intercorrelation matrix of all variables was obtained to ascertain that all predictors and mediators were significantly (*p* < 0.05) correlated with the cyberbullying variables.

A structural model with direct paths from all predictors to the mediator and the outcome (full model) was first fitted on the total sample data, and then separately for the female and male subgroups, using multigroup analysis. This model also controlled for participant age, which had a direct path to the outcome. The final multigroup model retained only those paths which overall and among the female and male subgroups were significant, constraining parameters such that these were similar across groups. Analyses were performed in the Mplus 8.4 software (Muthén and Muthén, 1998-2017) and based on full-information maximum-likelihood estimation (FIML), thus estimating the model parameters from all available data (4% missing values in the dataset). The robust maximum-likelihood estimator (MLR) was used to account for non-normality, which was the case for the current data. Concerning model fit, we report χ^2^ values (*p* > 0.05), the comparative fit index (CFI ≥ 0.90), the Tucker-Lewis index (TLI ≥ 0.95), the root mean square error of approximation (RMSEA < 0.08), and the standardized root mean square residual (SRMR < 0.08), with the utilized cut-off values for a good fit (see Kline, 2005; Hooper et al., 2008) provided in parentheses above.

## Results

### Preliminary Analyses

Cronbach α values indicated sufficient to good internal consistency for all scales and subscales (Table 1). Men had higher scores than women on most constructs, except on empathy (higher scores among women than men) and on emotional intelligence, vulnerable narcissism, and ECIPQ scores, for which no sex differences were apparent. Contrary to expectations, empathy neither correlated with the two cyberbullying variables in the total sample, nor with emotional intelligence, secondary psychopathy, or the two subscales of grandiose narcissism. Therefore, lacking significant associations with the outcome variables, empathy could not be used as a mediator in the present data and therefore was not included in the subsequent SEM analyses. Furthermore, no substantive correlations were observed between the two psychopathy factors and emotional intelligence.

### Structural Equation Models

The full model showed a good fit in the total sample, χ^2^ = 7.34, *df* = 8, *p* = 0.50, CFI = 1.000, TLI = 1.000, RMSEA = 0.000 [0.000, 0.041], SRMR = 0.010, as well as in the multigroup analysis, χ^2^ = 19.43, *df* = 17, *p* = 0.30, CFI = 0.996, TLI = 0.988, RMSEA = 0.020 [0.000, 0.053], SRMR = 0.021. In the multigroup model, the unstandardized loadings of the GAIT and ECIPQ scores on the latent cyberbullying factor were constrained to equality across groups to enforce weak measurement invariance. This ensured that the path coefficients of the predictors and the mediator to cyberbullying could be meaningfully compared between the groups. Standardized parameter estimates of the path coefficients in the two models are provided in Supplementary Table 1.

The final multigroup model (Figure 1) showed an improved data fit, as compared to the previous full model, χ^2^ = 32.35, *df* = 33, *p* = 0.50, CFI = 1.000, TLI = 1.000, RMSEA = 0.000 [0.000, 0.037], SRMR = 0.030. As the path of the Entitlement and Exhibitionism subscale to cyberbullying neither was significant among men nor women, this path was excluded from the final model.

As is evident from the numerical information in Figure 1, the model was mostly commensurable for women and men. All predictors, except Entitlement and Exhibitionism, had significant positive direct effects on cyberbullying. The strongest predictor was secondary psychopathy, closely followed by vulnerable narcissism, which, however, predicted cyberbullying only among men. All predictors, except secondary psychopathy and Entitlement and Exhibitionism among women, had additional indirect effects on cyberbullying via emotional intelligence. Entitlement and Exhibitionism had a weak indirect effect on cyberbullying as well, but only among men.

Except for the two subscales of grandiose narcissism, all indirect effects were associated with net increases in cyberbullying, i.e., pointed into the same direction as the direct effects, because the predictors were negatively correlated with emotional intelligence, which in turn was negatively correlated with cyberbullying. However, this direction was reversed for the two grandiose narcissism subscales (Entitlement and Exhibitionism only among men), which were *positively* associated with emotional intelligence. That is, higher scores on these subscales corresponded to higher scores on emotional intelligence, which in turn *decreased* cyberbullying behavior. However, the total effect of Leadership (combining direct and indirect effects) still was positive (0.09 among men, 0.12 among women, *p*s ≤ 0.020).

In total, the model explained 45% (men) and 36% (women; both *p*s < 0.001) of the variance of latent cyberbullying. Participant age was negatively associated with cyberbullying among men and women alike.

## Discussion

The objective of the study was to examine the mutual associations between the Dark Triad traits, emotional intelligence, empathy, and cyberbullying, and to control for age effects and possible sex differences. The results suggested that the Dark Triad traits are associated with more self-reported cyberbullying behavior. However, only emotional intelligence, but not empathy, appeared to play a mediating role for these associations. Further, some differences between men and women emerged, concerning the associations of grandiose and vulnerable narcissism with cyberbullying. In all, our results suggest that high emotional intelligence is associated with less cyberbullying and is not used as a means to harm others (cf. Sutton et al., 2001; Baughman et al., 2012; Nagler et al., 2014; Vonk et al., 2015; Davis and Nichols, 2016). Quite the contrary, dark personality traits were mostly associated with low emotional intelligence. For grandiose narcissism, which was associated with high emotional intelligence, there appeared to be even buffering effects of emotional intelligence on cyberbullying. Our results further suggest that cyberbullying decreases with age, which is in line with previous findings (Ionut-Dorin, 2017).

Secondary psychopathy, but also primary psychopathy, was associated with more cyberbullying among both men and women, thus corroborating previous results. With regards to secondary psychopathy, our findings are in line with Baughman et al. (2012), who stipulated that psychopathy predicts aggressive behavior, and with Goodboy and Martin (2015), who found psychopathy to be the only significant predictor for visually and textually based cyberbullying. Individuals scoring high in secondary psychopathy have been described as highly antisocial and impulsive (Karpman, 1941; Bechara et al., 2000), whilst cyberbullies as individuals with behavioral problems (Aboujaoude et al., 2015). On the other hand, individuals high in primary psychopathy appear to be more adjusted and strategic than secondary psychopaths (Gao and Raine, 2010) and tend to use more indirect aggression (Vaillancourt and Sunderani, 2011), which also is the predilected form of aggression among women (Gavin and Porter, 2014). The associations of primary and secondary psychopathy in the present study thus highlight that cyberbullying may either be an impulsive or a strategic form of aggression, depending on the perpetrator’s profile. This should be followed up in future research.

Only primary, but not secondary, psychopathy was associated with emotional intelligence in the present study. Yet, this association only became significant in the structural equation model and was not particularly strong. Jauk et al. (2016) found negative associations between psychopathy and emotional intelligence. However, Ali et al. (2009) and Szabó and Bereczkei (2017), did not observe associations with primary psychopathy. These differences between our results and these prior related ones might be due to different scales used to measure psychopathy (Copestake et al., 2013; Megías et al., 2018).

The results regarding the associations between grandiose narcissism and cyberbullying mirror the results by Baughman et al. (2012) on indirect bullying. Our findings thus contest Kircaburun et al. (2018), who found that role of narcissism for relational aggression diminishes when psychopathy is considered. Furthermore, our results suggest that vulnerable narcissism is also linked to cyberbullying behavior, particularly among men, for whom a direct path of vulnerable narcissism to cyberbullying behavior was also apparent. This is in line with findings emphasizing a link between vulnerable narcissism and hostility (Hyatt et al., 2018) and with more covert and indirect ways of aggression (Okada, 2010). The negative association between vulnerable narcissism and emotional intelligence in the current study parallels findings by Zajenkowski et al. (2018), who argued that vulnerable narcissists might be less positively biased than grandiose narcissists and might evaluate their emotional intelligence abilities as being low. The role of vulnerable narcissism for antisocial and aggressive behavior needs more study and, as suggested by the present study, in this context possible sex differences need to be examined as well.

The positive association between grandiose narcissism and emotional intelligence is supported by Rogosch and Cicchetti (2004) and Vonk et al. (2015). Yet, our data suggest that this association may also mitigate the otherwise negative effects of narcissism when it comes to cyberbullying. Emotional intelligence may provide some buffering effects specifically for grandiose narcissism. This is a new finding, which should be followed up in future research. However, there is also evidence that individuals high in grandiose narcissism overestimate their emotional abilities (Jauk et al., 2016; Zajenkowski et al., 2018). Hence, it may be necessary to not only investigate trait emotional intelligence in future studies, but also ability emotional intelligence, which may provide a more accurate account of individuals’ actual competences.

Contrary to some previous findings (Goodboy and Martin, 2015), Machiavellianism was both directly and indirectly associated with cyberbullying. Yet, Machiavellianism has previously been reported to uniquely predict bullying both outside (van Geel et al., 2017) and inside the cyberspace (Kircaburun et al., 2018) and to be negatively associated with emotional intelligence (Ali et al., 2009; Petrides et al., 2011; Czibor et al., 2017). Psychopathy may account for callousness and manipulation, which also are present in Machiavellianism and narcissism (Jones and Figueredo, 2013; Bertl et al., 2017). Hence, once again, differences between previous results and the present findings may be due to different scales used and whether these show item-content overlap with related traits or not. The MACH^∗^ used by us has better discriminant validity than the MACH-IV, from which instrument it has been derived (Rauthmann, 2013).

Emotional intelligence appears to be a negative predictor for cyberbullying. This corresponds to findings by Baroncelli and Ciucci (2014), Ang et al. (2017), and Beltrán-Catalán et al. (2018). However, the fact that no association was found between cyberbullying and empathy is surprising, as Kowalski et al. (2018) reported a negative correlation with affective empathy, and Ang et al. (2017) that both affective and perspective taking negatively predicted cyberbullying. One possible explanation might be that the associations between affective empathy and cyberbullying are partially mediated and moderated by normative beliefs about aggression (Ang et al., 2017). Yet, it might also have to do with validity aspects of the IRI, which has been characterized as mainly being a measure of perspective taking (Maibom, 2014).

Even though cyberbullying and empathy were not associated in the present data, we still observed correlations between the Dark Triad traits and empathy. Specifically, primary psychopathy was associated with a lack of empathy, as was Machiavellianism, which is in line with previous findings (Karpman, 1941; Szabó and Bereczkei, 2017; Monaghan et al., 2018). Secondary psychopathy did not correlate with empathy, which contradicts a previous report by Guttman and Laporte (2002), but is supported by findings of Szabó and Bereczkei (2017).

The fact that we did not find any relevant associations between grandiose narcissism and empathy mirrors previous findings by Findley and Ojanen (2013), but is inconsistent with another study (Hepper et al., 2014). The positive association of vulnerable narcissism with empathy in the present study is a new finding. A speculative explanation may be provided through the assumption that fear of other people’s judgment incites more perspective taking among individuals high in vulnerable narcissism. More research is needed here as well.

The negative correlation between empathy and age is in line with studies showing that even though affective empathy might increase with older age, perspective taking declines, as it becomes more difficult to recognize emotions, which requires more effort and is less automatic (Khanjani et al., 2015). In addition, it has been reported that self-other differentiation (Studer et al., 2009) and perspective taking (Zhang et al., 2013) decrease with age. Since the IRI mainly measures perspective taking (Maibom, 2014), our results are in line with reports of declining effects of perspective taking with age as well.

### Study Strengths and Limitations, and Directions for Future Research

Strengths of the present study comprise the usage of a large and diverse community sample and utilizing comprehensive measures of the Dark Triad personality traits for assessment, along with structural equation modeling for analysis. The longer scales administered here differentiated between the Dark Triad facets and provided more information than global Dark Triad measures. However, despite being large and diverse, the sample was not representative of the general population, as educational levels on average were too high and ages too young. Yet, the sample still comprised all age groups and educational levels present in the general population and was nearly balanced for sex, which has not been the case in most other studies in this field of research. Considering that extant studies sampled mostly female, undergraduate, and much younger individuals, the present database thus constitutes an improvement.

A second study limitation concerns the empathy measure administered here. According to Paulus (2009), the IRI measures empathy in fictive and real situations (cf. Tran et al., 2013). However, as pointed out by Maibom (2014), several of the IRI items might be more adequate to measure sympathy and perspective taking in general than to specifically measure (affective) empathy. A third limitation concerns the sample reliabilities of some of the scales used. The reliability of the primary psychopathy subscale of the SRP-III and of the HSNS appeared low and in need of improvement. Finally, item and scale distributions were skewed, which likely is a consequence of social desirability effects which remain a pervasive problem in this field of research (Muris et al., 2017), as is the reliance on cross-sectional designs and self-reports as the data level. Specifically, self-reports, and observed associations, could be subject to method variance and response sets (e.g., socially desirable or mischievous responding).

The present study replicated several findings from prior related research, but also yielded novel and more differentiated evidence, all of which may provide a better understanding of the nexus of the constructs scrutinized here and may fruitfully spur further inquiry along these lines. Future research would benefit from replicating the current set of findings and those of extant studies, in order to provide more clarity on this theme. Additionally, future studies should further improve on the scales and methods of measurement in this thematic area. More generally, future research could beneficially exploit longitudinal designs and other data levels (e.g., experience sampling approaches; and behavioral, group-based, implicit, or unobtrusive data sources). Altogether, this would raise the probability of clear, replicable, and reliable results, on which eventually prevention programs for counteracting cyberbullying, informed by personality science, and their implementation in schools and the workplace, could be based.

### Conclusion

This study provides detailed insights into important facilitating factors for cyberbullying and into the internal structure and the inner workings of dark personality traits. Psychopathy, narcissism, and Machiavellianism all are associated with cyberbullying behavior, in a broadly similar fashion among both women and men. Emotional intelligence partly mediates these associations. Sex-specific patterns in the associations of Dark Triad traits with cyberbullying are particularly observable with regards to grandiose and vulnerable narcissism. Future research should investigate cyberbullies’ profiles with regard to primary and secondary psychopathy, sex differences in antisocial and aggressive behavior among individuals high in vulnerable narcissism, and potentially buffering effects of emotional intelligence on grandiose narcissism. Further improvements on the measurement of dark personality traits are also indicated.

## Data Availability Statement

All study data can be accessed at https://doi.org/10.6084/m9.figshare.13636379.v1.

## Ethics Statement

Ethical review and approval was not required for the study on human participants in accordance with the local legislation and institutional requirements. The patients/participants provided their written informed consent to participate in this study.

## Author Contributions

UT conceived of the original research idea, designed the study, and supervised the data collection. ES and UT performed the statistical analysis. ES contributed to theory and methodology and wrote the first draft of the manuscript, with assistance and contributions from UT and MV. UT and MV provided important intellectual content in revising the manuscript. All authors have reviewed and approved the final manuscript.

## Conflict of Interest

The authors declare that the research was conducted in the absence of any commercial or financial relationships that could be construed as a potential conflict of interest.

## References

[B1] Aboujaoude E. Savage M. W. Starcevic V. Salame W. O. (2015). Cyberbullying: review of an old problem gone viral. *J. Adolesc. Health* 57 10–18. 10.1016/j.jadohealth.2015.04.011 26095405

[B2] Ackerman R. A. Witt E. A. Donnellan M. B. Trzesniewski K. H. Robins R. W. Kashy D. A. (2011). What does the narcissistic personality inventory really measure? *Assessment* 18 67–87. 10.1177/1073191110382845 20876550

[B3] Ali F. Amorim I. S. Chamorro-Premuzic T. (2009). Empathy deficits and trait emotional intelligence in psychopathy and Machiavellianism. *Pers. Individ. Differ.* 47 758–762. 10.1016/j.paid.2009.06.016

[B4] Ang R. P. Li X. Seah S. L. (2017). The role of normative beliefs about aggression in the relationship between empathy and cyberbullying. *J. Cross Cult. Psychol.* 48 1138–1152. 10.1177/0022022116678928

[B5] Bacon A. M. Regan L. (2016). Manipulative relational behaviour and delinquency: sex differences and links with emotional intelligence. *J. Forens. Psychiatr. Psychol.* 27 331–348. 10.1080/14789949.2015.1134625

[B6] Baroncelli A. Ciucci E. (2014). Unique effects of different components of trait emotional intelligence in traditional bullying and cyberbullying. *J. Adolesc.* 37 807–815. 10.1016/j.adolescence.2014.05.009 25086457

[B7] Baughman H. M. Dearing S. Giammarco E. Vernon P. A. (2012). Relationships between bullying behaviours and the dark triad: a study with adults. *Pers. Individ. Differ.* 52 571–575. 10.1016/j.paid.2011.11.020

[B8] Bechara A. Tranel D. Damasio H. (2000). Characterization of the decision-making deficit of patients with ventromedial prefrontal cortex lesions. *Brain* 123 2189–2202. 10.1093/brain/123.11.2189 11050020

[B9] Behling O. Law K. S. (2000). *Translating Questionnaires and Other Research Instruments: Problems and Solutions.* Thousand Oaks, CA: Sage.

[B10] Beller J. Bosse S. (2017). Machiavellianism has a dimensional latent structure: results from taxometric analyses. *Pers. Individ. Differ.* 113 57–62. 10.1016/j.paid.2017.03.014

[B11] Beltrán-Catalán M. Zych I. Ortega-Ruiz R. (2018). Victimisation through bullying and cyberbullying: emotional intelligence, severity of victimisation and technology use in different types of victims. *Psicothema* 30 183–188. 10.7334/psicothema2017.313 29694319

[B12] Bertl B. Pietschnig J. Tran U. S. Stieger S. Voracek M. (2017). More or less than the sum of its parts? Mapping the Dark Triad of personality onto a single Dark Core. *Pers. Individ. Differ.* 114 140–144. 10.1016/j.paid.2017.04.002

[B13] Boulton M. J. Smith P. K. (1994). Bully/victim problems in middle-school children: stability, self-perceived competence, peer perceptions and peer acceptance. *Br. J. Dev. Psychol.* 12 315–329. 10.1111/j.2044-835X.1994.tb00637.x

[B14] Buckels E. E. Trapnell P. D. Paulhus D. L. (2014). Trolls just want to have fun. *Pers. Individ. Differ.* 67 97–102. 10.1016/j.paid.2014.01.016

[B15] Campbell K. W. Miller J. D. Buffardi L. E. (2010). The United States and the “culture of narcissism”: an examination of perceptions of national character. *Soc. Psychol. Pers. Sci.* 1 222–229. 10.1177/1948550610366878

[B16] Christie R. Geis F. L. Festinger L. Schachter S. (1970). *Studies in Machiavellianism.* Cambridge, MA: Academic Press, 10.1016/C2013-0-10497-7

[B17] Cleckley H. M. (1988). *The Mask of Sanity*, 5th Edn, Maryland Heights, MO: Mosby.

[B18] Copestake S. Gray N. S. Snowden R. J. (2013). Emotional intelligence and psychopathy: a comparison of trait and ability measures. *Emotion* 13 691–702. 10.1037/a0031746 23527501

[B19] Czibor A. Szabo Z. P. Jones D. N. Zsido A. N. Paal T. Szijjarto L. (2017). Male and female face of Machiavellianism: opportunism or anxiety? *Pers. Individ. Differ.* 117 221–229. 10.1016/j.paid.2017.06.002

[B20] Davis M. H. (1980). A multidimensional approach to individual differences in empathy. *JSAS Catal. Select. Doc. Psychol.* 10:85.

[B21] Davis S. K. Nichols R. (2016). Does emotional intelligence have a “dark” side? A review of the literature. *Front. Psychol.* 7:1316. 10.3389/fpsyg.2016.01316 27625627 PMC5003940

[B22] Del Rey R. Casas J. A. Ortega-Ruiz R. Schultze-Krumbholz A. Scheithauer H. Smith P. (2015). Structural validation and cross-cultural robustness of the European Cyberbullying intervention project questionnaire. *Comput. Hum. Behav.* 50 141–147. 10.1016/j.chb.2015.03.065

[B23] Dickinson K. A. Pincus A. L. (2003). Interpersonal analysis of grandiose and vulnerable narcissism. *J. Pers. Disord.* 17 188–207. 10.1521/pedi.17.3.188.22146 12839099

[B24] Fanti K. A. Kimonis E. R. (2012). Bullying and victimization: the role of conduct problems and psychopathic traits. *J. Res. Adolesc.* 22 617–631. 10.1111/j.1532-7795.2012.00809.x

[B25] Findley D. Ojanen T. (2013). Agentic and communal goals in early adulthood: associations with narcissism, empathy, and perceptions of self and others. *Self Ident.* 12 504–526. 10.1080/15298868.2012.694660

[B26] Gao Y. Raine A. (2010). Successful and unsuccessful psychopaths: a neurobiological model. *Behav. Sci. Law* 28 194–210. 10.1002/bsl.924 20422645

[B27] Gavin H. Porter T. (2014). *Female Aggression.* Hoboken, NJ: Wiley.

[B28] Gibb Z. G. Devereux P. G. (2014). Who does that anyway? Predictors and personality correlates of cyberbullying in college. *Comput. Hum. Behav.* 38 8–16. 10.1016/j.chb.2014.05.009

[B29] Gillespie A. A. (2006). Cyber-bullying and harassment of teenagers: the legal response. *J. Soc. Welfare Fam. Law* 28 123–136. 10.1080/09649060600973772

[B30] Goodboy A. K. Martin M. M. (2015). The personality profile of a cyberbully: examining the Dark Triad. *Comput. Hum. Behav.* 49 1–4. 10.1016/j.chb.2015.02.052

[B31] Grijalva E. Newman D. A. Tay L. Donnellan M. B. Harms P. D. Robins R. W. (2015). Gender differences in narcissism: a meta-analytic review. *Psychol. Bull.* 141 261–310. 10.1037/a0038231 25546498

[B32] Guttman H. Laporte L. (2002). Alexithymia, empathy, and psychological symptoms in a family context. *Comprehens. Psychiatry* 43 448–455. 10.1053/comp.2002.35905 12439832

[B33] Hart S. D. Hare R. D. Harpur T. J. (1992). “The Psychopathy Checklist—Revised (PCL-R),” in *Advances in Psychological Assessment*, eds Rosen J. C. McReynolds P. (Cham: Springer), 103–130. 10.1007/978-1-4757-9101-3_4

[B34] Hendin H. M. Cheek J. M. (1997). Assessing hypersensitive narcissism: a reexamination of Murray’s narcism scale. *J. Res. Pers.* 31 588–599. 10.1006/jrpe.1997.2204

[B35] Hepper E. G. Hart C. M. Meek R. Cisek S. Sedikides C. (2014). Narcissism and empathy in young offenders and non-offenders: narcissism in offenders. *Eur. J. Pers.* 28 201–210. 10.1002/per.1939

[B36] Hicks B. M. Carlson M. D. Blonigen D. M. Patrick C. J. Iacono W. G. MGue M. (2012). Psychopathic personality traits and environmental contexts: differential correlates, gender differences, and genetic mediation. *Pers. Disord. Theory Res. Treat.* 3 209–227. 10.1037/a0025084 22452762 PMC3387315

[B37] Hooper D. Coughlan J. Mullen M. R. (2008). Structural equation modeling: guidelines for determining model fit. *Electron. J. Bus. Res. Methods* 6 53–60.

[B38] Hyatt C. S. Sleep C. E. Lynam D. R. Widiger T. A. Campbell W. K. Miller J. D. (2018). Ratings of affective and interpersonal tendencies differ for grandiose and vulnerable narcissism: a replication and extension of Gore and Widiger (2016). *J. Pers.* 86 422–434. 10.1111/jopy.12325 28509415

[B39] Ionut-Dorin S. (2017). Age differences and preferences in online behavior. How ageing and digital connectedness are reflected in current research regarding the use of social media. *eLearn. Softw. Educ. Conf.* 2 624–631. 10.12753/2066-026X-17-173

[B40] Jauk E. Freudenthaler H. H. Neubauer A. C. (2016). The Dark Triad and trait versus ability emotional intelligence: emotional darkness differs between women and men. *J. Individ. Differ.* 37 112–118. 10.1027/1614-0001/a000195

[B41] Jauk E. Kaufman S. B. (2018). The higher the score, the darker the core: the nonlinear association between grandiose and vulnerable narcissism. *Front. Psychol.* 9:1305. 10.3389/fpsyg.2018.01305 30150950 PMC6088174

[B42] Jonason P. K. Webster G. D. (2010). The dirty dozen: a concise measure of the Dark Triad. *Psychol. Assess.* 22 420–432. 10.1037/a0019265 20528068

[B43] Jones D. N. Figueredo A. J. (2013). The core of darkness: uncovering the heart of the Dark Triad. *Eur. J. Pers.* 27 521–531. 10.1002/per.1893

[B44] Jones D. N. Paulhus D. L. (2014). Introducing the Short Dark Triad (SD3): a brief measure of dark personality traits. *Assessment* 21 28–41. 10.1177/1073191113514105 24322012

[B45] Jung H. S. Yoon H. H. (2012). The effects of emotional intelligence on counterproductive work behaviors and organizational citizen behaviors among food and beverage employees in a deluxe hotel. *Intern. J. Hosp. Manag.* 31 369–378. 10.1016/j.ijhm.2011.06.008

[B46] Karpman B. (1941). On the need of separating psychopathy into two distinct clinical types: the symptomatic and the idiopathic. *J. Crim. Psychopathol.* 3 112–137.

[B47] Keskin H. Akgün A. E. Ayar H. Kayman Ş (2016). Cyberbullying victimization, counterproductive work behaviours and emotional intelligence at workplace. *Proc. Soc. Behav. Sci.* 235 281–287. 10.1016/j.sbspro.2016.11.031

[B48] Khanjani Z. Mosanezhad Jeddi E. Hekmati I. Khalilzade S. Etemadi Nia M. Andalib M. (2015). Comparison of cognitive empathy, emotional empathy, and social functioning in different age groups: Empathy and social functioning. *Austr. Psychol.* 50 80–85. 10.1111/ap.12099

[B49] Kircaburun K. Jonason P. K. Griffiths M. D. (2018). The Dark Tetrad traits and problematic social media use: The mediating role of cyberbullying and cyberstalking. *Pers. Individ. Differ.* 135 264–269. 10.1016/j.paid.2018.07.034

[B50] Kline R. B. (2005). *Methodology in the Social Sciences: Principles and Practice of Structural Equation Modeling*, 2nd Edn, New York, NY: Guilford.

[B51] Knight N. M. Dahlen E. R. Bullock-Yowell E. Madson M. B. (2018). The HEXACO model of personality and Dark Triad in relational aggression. *Pers. Individ. Differ.* 122 109–114. 10.1016/j.paid.2017.10.016

[B52] Kowalski R. M. Limber S. P. McCord A. (2018). A developmental approach to cyberbullying: prevalence and protective factors. *Aggress. Viol. Behav.* 45 20–32. 10.1016/j.avb.2018.02.009

[B53] Lander G. C. Lutz-Zois C. J. Rye M. S. Goodnight J. A. (2012). The differential association between alexithymia and primary versus secondary psychopathy. *Pers. Individ. Differ.* 52 45–50. 10.1016/j.paid.2011.08.027

[B54] Lilienfeld S. O. Andrews B. P. (1996). Development and preliminary validation of a self-report measure of psychopathic personality traits in noncriminal population. *J. Pers. Assess.* 66 488–524. 10.1207/s15327752jpa6603_3 8667144

[B55] Lowry P. B. Zhang J. Wang C. Siponen M. (2016). Why do adults engage in cyberbullying on social media? An integration of online disinhibition and deindividuation effects with the social structure and social learning model. *Inform. Syst. Res.* 27 962–986. 10.1287/isre.2016.0671 19642375

[B56] Maibom H. L. (2014). “Without fellow feeling,” in *Being Amoral: Psychopathy and Moral Incapacity*, ed. Schramme T. (Cambridge, MA: MIT Press), 91–114. 10.7551/mitpress/9780262027915.003.0004

[B57] Matthews E. (2014). “Psychopathy and moral rationality,” in *Being Amoral: Psychopathy and Moral Incapacity*, ed. Schramme T. (Cambridge, MA: MIT Press), 71–89. 10.7551/mitpress/9780262027915.003.0003

[B58] Mavroveli S. Petrides K. V. Rieffe C. Bakker F. (2007). Trait emotional intelligence, psychological well-being and peer-rated social competence in adolescence. *Br. J. Dev. Psychol.* 25 263–275. 10.1348/026151006X118577

[B59] Megías A. Gómez-Leal R. Gutiérrez-Cobo M. J. Cabello R. Fernández-Berrocal P. (2018). The relationship between trait psychopathy and emotional intelligence: a meta-analytic review. *Neurosci. Biobehav. Rev.* 84 198–203. 10.1016/j.neubiorev.2017.12.003 29217464

[B60] Miller J. D. Dir A. Gentile B. Wilson L. Pryor L. R. Campbell W. K. (2010). Searching for a vulnerable Dark Triad: comparing factor 2 psychopathy, vulnerable narcissism, and borderline personality disorder: vulnerable Dark triad. *J. Pers.* 78 1529–1564. 10.1111/j.1467-6494.2010.00660.x 20663024

[B61] Miller J. D. Hoffman B. J. Gaughan E. T. Gentile B. Maples J. Campbell W. K. (2011). Grandiose and vulnerable narcissism: a nomological network analysis. *J. Pers.* 79 1013–1042. 10.1111/j.1467-6494.2010.00711.x 21204843

[B62] Monaghan C. Bizumic B. Sellbom M. (2018). Nomological network of two-dimensional Machiavellianism. *Pers. Individ. Differ.* 130 161–173. 10.1016/j.paid.2018.03.047

[B63] Muris P. Merckelbach H. Otgaar H. Meijer E. (2017). The malevolent side of human nature: a meta-analysis and critical review of the literature on the Dark Triad (narcissism, Machiavellianism, and psychopathy). *Perspect. Psychol. Sci.* 12 183–204. 10.1177/1745691616666070 28346115

[B64] Muthén L. K. Muthén B. O. (1998-2017). *Mplus User’s Guide*, 8th Edn, Los Angeles, CA: Muthén & Muthén.

[B65] Nagler U. K. J. Reiter K. J. Furtner M. R. Rauthmann J. F. (2014). Is there a “dark intelligence”? Emotional intelligence is used by dark personalities to emotionally manipulate others. *Pers. Individ. Differ.* 65 47–52. 10.1016/j.paid.2014.01.025

[B66] Okada R. (2010). The relationship between vulnerable narcissism and aggression in Japanese undergraduate students. *Pers. Individ. Differ.* 49 113–118. 10.1016/j.paid.2010.03.017

[B67] Pabian S. De Backer C. J. S. Vandebosch H. (2015). Dark Triad personality traits and adolescent cyber-aggression. *Pers. Individ. Differ.* 75 41–46. 10.1016/j.paid.2014.11.015

[B68] Parton D. M. Ent M. R. (2018). Vulnerable narcissism predicts greater spiteful punishment of a third-party transgressor. *J. Res. Pers.* 76 150–153. 10.1016/j.jrp.2018.08.005

[B69] Paulhus D. L. Neumann C. S. Hare R. D. Williams K. Hemphill J. (2016). *Self-Report Psychopathy Scale^TM^*, 4th Edn, Toronto: Multi-Health Systems.

[B70] Paulhus D. L. Williams K. M. (2002). The Dark Triad of personality: narcissism, machiavellianism, and psychopathy. *J. Res. Pers.* 36 556–563. 10.1016/S0092-6566(02)00505-6

[B71] Paulus C. (2009). *Der Saarbrücker Persönlichkeitsfragebogen SPF (IRI) zur Messung von Empathie: Psychometrische Evaluation der deutschen Version des Interpersonal Reactivity Index.* Available online at: http://hdl.handle.net/20.500.11780/3343 (accessed April 4, 2019).

[B72] Petrides K. V. (2009). “Psychometric properties of the trait emotional intelligence questionnaire (TEIQue),” in *Assessing Emotional Intelligence*, eds Parker J. D. A. Saklofske D. H. Stough C. (Berlin: Springer), 85–101. 10.1007/978-0-387-88370-0_5

[B73] Petrides K. V. Furnham A. (2006). The role of trait emotional intelligence in a gender-specific model of organizational variables. *J. Appl. Soc. Psychol.* 36 552–569. 10.1111/j.0021-9029.2006.00019.x

[B74] Petrides K. V. Vernon P. A. Schermer J. A. Veselka L. (2011). Trait emotional intelligence and the Dark Triad traits of personality. *Twin Res. Hum. Genet.* 14 35–41. 10.1375/twin.14.1.35 21314254

[B75] Poythress N. G. Hall J. R. (2011). Psychopathy and impulsivity reconsidered. *Aggress. Viol. Behav.* 16 120–134. 10.1016/j.avb.2011.02.003

[B76] Raskin R. N. Hall C. S. (1979). A narcissistic personality inventory. *Psychol. Rep.* 45:590. 10.2466/pr0.1979.45.2.590 538183

[B77] Rauthmann J. F. (2013). Investigating the MACH-IV with item response theory and proposing the trimmed MACH^∗^. *J. Pers. Assessm.* 95 388–397. 10.1080/00223891.2012.742905 23186231

[B78] Rauthmann J. F. Will T. (2011). Proposing a multidimensional Machiavellianism conceptualization. *Soc. Behav. Pers.* 39 391–403. 10.2224/sbp.2011.39.3.391

[B79] Rogosch F. A. Cicchetti D. (2004). Child maltreatment and emergent personality organization: Perspectives from the five-factor model. *J. Abnorm. Child Psychol.* 32 123–145. 10.1023/B:JACP.0000019766.47625.40 15164856

[B80] Salovey P. Mayer J. D. (1990). Emotional intelligence. *Imag. Cogn. Pers.* 9 185–211. 10.2190/DUGG-P24E-52WK6CDG 22612255

[B81] Schokman C. Downey L. A. Lomas J. Wellham D. Wheaton A. Simmons N. (2014). Emotional intelligence, victimisation, bullying behaviours and attitudes. *Learn. Individ. Differ.* 36 194–200. 10.1016/j.lindif.2014.10.013

[B82] Schultze-Krumbholz A. Scheithauer H. (2011). *Der Berlin Cyberbullying-Cybervictimisation Questionnaire (BCyQ).* Berlin: Freie Universität Berlin.

[B83] Skeem J. Johansson P. Andershed H. Kerr M. Louden J. E. (2007). Two subtypes of psychopathic violent offenders that parallel primary and secondary variants. *J. Abnorm. Psychol.* 116 395–409. 10.1037/0021-843X.116.2.395 17516770

[B84] Spangenberg L. Romppel M. Bormann B. Hofmeister D. Brähler E. Strauss B. (2013). A short version of the Narcissistic Personality Inventory (NPI-15): Dimensionality and psychometric properties in a representative sample of the German population. *Psychother. Psychosom. Med. Psychol.* 63 341–347. 10.1055/s-0032-1333288 23475762

[B85] Studer J. Mella-Barraco N. Labouvie-Vief G. (2009). “Age differences in empathy: Self-other differentiation becomes more difficult with age,” in *Proceedings of the 49th Annual Meeting of the Society-for-Psychophysiological-Research Conference*, Berlin.

[B86] Sutton J. Smith P. K. Swettenham J. (2001). Bullying and ‘theory of mind’: a critique of the ‘social skills deficit’ view of anti-social behaviour. *Soc. Dev.* 8 117–127. 10.1111/1467-9507.00083

[B87] Szabó E. Bereczkei T. (2017). Different paths to different strategies? Unique associations among facets of the Dark Triad, empathy, and trait emotional intelligence. *Adv. Cogn. Psychol.* 13 306–313. 10.5709/acp-0230-7 29362646 PMC5771371

[B88] Tokunaga R. S. (2010). Following you home from school: a critical review and synthesis of research on cyberbullying victimization. *Comput. Hum. Behav.* 26 277–287. 10.1016/j.chb.2009.11.014

[B89] Tran U. S. Bertl B. Kossmeier M. Pietschnig J. Stieger S. Voracek M. (2018). “I’ll teach you differences”: taxometric analysis of the Dark Triad, trait sadism, and the Dark Core of personality. *Pers. Individ. Differ.* 126 19–24. 10.1016/j.paid.2018.01.015

[B90] Tran U. S. Laireiter A. R. Neuner C. Schmitt D. P. Leibetseder M. Szente-Voracek S. L. (2013). Factorial structure and convergent and discriminant validity of the E (Empathy) Scale. *Psychol. Rep.* 113 441–463. 10.2466/03.02.PR0.113x20z9 24597440

[B91] Vaillancourt T. Sunderani S. (2011). Psychopathy and indirect aggression: the roles of cortisol, sex, and type of psychopathy. *Brain Cogn.* 77 170–175. 10.1016/j.bandc.2011.06.009 21855201

[B92] van Geel M. Goemans A. Toprak F. Vedder P. (2017). Which personality traits are related to traditional bullying and cyberbullying? A study with the Big Five, Dark Triad and sadism. *Pers. Individ. Differ.* 106 231–235. 10.1016/j.paid.2016.10.063

[B93] Veselka L. Giammarco E. A. Vernon P. A. (2014). The Dark Triad and the seven deadly sins. *Pers. Individ. Differ.* 67 75–80. 10.1016/j.paid.2014.01.055

[B94] Vonk J. Zeigler-Hill V. Ewing D. Mercer S. Noser A. E. (2015). Mindreading in the dark: dark personality features and theory of mind. *Pers. Individ. Differ.* 87 50–54. 10.1016/j.paid.2015.07.025

[B95] Wallace H. M. Scheiner B. R. M. Grotzinger A. (2016). Grandiose narcissism predicts willingness to behave badly, without proportional tolerance for others’ bad behavior. *Curr. Psychol.* 35 234–243. 10.1007/s12144-016-9410-x

[B96] Wang X. Lei L. Liu D. Hu H. (2016). Moderating effects of moral reasoning and gender on the relation between moral disengagement and cyberbullying in adolescents. *Pers. Individ. Differ.* 98 244–249. 10.1016/j.paid.2016.04.056

[B97] Yildirim B. O. Derksen J. J. L. (2013). Systematic review, structural analysis, and new theoretical perspectives on the role of serotonin and associated genes in the etiology of psychopathy and sociopathy. *Neurosci. Biobehav. Rev.* 37 1254–1296. 10.1016/j.neubiorev.2013.04.009 23644029

[B98] Zajenkowski M. Maciantowicz O. Szymaniak K. Urban P. (2018). Vulnerable and grandiose narcissism are differentially associated with ability and trait emotional intelligence. *Front. Psychol.* 9:1606. 10.3389/fpsyg.2018.01606 30210418 PMC6120976

[B99] Zhang X. Fung H. H. Stanley J. T. Isaacowitz D. M. Ho M. Y. (2013). Perspective taking in older age revisited: a motivational perspective. *Dev. Psychol.* 49 1848–1858. 10.1037/a0031211 23276131

