# Supplementary material for: The Nexus of the Dark Triad Personality Traits With Cyberbullying, Empathy, and Emotional Intelligence: A Structural-Equation Modeling Approach
Source: Front Psychol. 2021 Jun 4;12:659282. doi: 10.3389/fpsyg.2021.659282 (PMC8211728; doi:10.3389/fpsyg.2021.659282)
Supplement: Supplementary file 1 [file Table_1.DOCX]

Supplementary Material

**Table S1.** Standardized regression coefficients, standard errors, and *p* values for the direct paths from the mediator and the predictors to cyberbullying, and the predictors to the mediator in the full model.

|  | Multigroup model | | | | | |  | | |
| --- | --- | --- | --- | --- | --- | --- | --- | --- | --- |
|  | Men | | | Women | | | Total sample | | |
|  | Beta | *SE* | *p* | Beta | *SE* | *p* | Beta | *SE* | *p* |
| Direct paths to cyberbullying |  |  |  |  |  |  |  |  |  |
| Emotional intelligence | -0.17 | 0.06 | .007 | -0.15 | 0.07 | .034 | -0.15 | 0.05 | .001 |
| Primary psychopathy | 0.11 | 0.08 | .154 | 0.15 | 0.07 | .023 | 0.17 | 0.05 | .001 |
| Secondary psychopathy | 0.29 | 0.06 | <.001 | 0.16 | 0.08 | .041 | 0.26 | 0.05 | <.001 |
| Leadership | 0.25 | 0.07 | .001 | 0.14 | 0.09 | .103 | 0.22 | 0.05 | <.001 |
| Entitlement and Exhibitionism | -0.16 | 0.07 | .032 | 0.03 | 0.11 | .750 | -0.09 | 0.06 | .129 |
| Vulnerable narcissism | 0.18 | 0.06 | .004 | -0.03 | 0.07 | .720 | 0.08 | 0.04 | .076 |
| Machiavellianism | 0.11 | 0.06 | .058 | 0.15 | 0.06 | .019 | 0.16 | 0.04 | <.001 |
| Control variable age | -0.31 | 0.05 | <.001 | -0.23 | 0.05 | <.001 | -0.26 | 0.03 | <.001 |
|  |  |  |  |  |  |  |  |  |  |
| Direct paths to emotional intelligence |  |  |  |  |  |  |  |  |  |
| Primary psychopathy | -0.17 | 0.06 | .005 | -0.10 | 0.06 | .081 | -0.13 | 0.04 | .002 |
| Secondary psychopathy | -0.01 | 0.06 | .836 | -0.02 | 0.06 | .781 | -0.01 | 0.04 | .820 |
| Leadership | 0.32 | 0.07 | <.001 | 0.31 | 0.07 | <.001 | 0.32 | 0.05 | <.001 |
| Entitlement and Exhibitionism | 0.15 | 0.07 | .022 | 0.05 | 0.08 | .564 | 0.10 | 0.05 | .047 |
| Vulnerable narcissism | -0.40 | 0.05 | <.001 | -0.35 | 0.05 | <.001 | -0.38 | 0.03 | <.001 |
| Machiavellianism | -0.18 | 0.06 | .001 | -0.20 | 0.05 | <.001 | -0.18 | 0.04 | <.001 |
